# Supplementary material for: Chromothripsis during telomere crisis is independent of NHEJ, and consistent with a replicative origin
Source: Genome Res. 2019 May;29(5):737–49. doi: 10.1101/gr.240705.118 (PMC6499312; doi:10.1101/gr.240705.118)
Supplement: Supplemental Material [file supp_gr.240705.118_Supplemental_file_1.zip › contigs/annotated_contigs/DB69/contig.2.DB69_length_590_mean_cov_11.793220339.docx]

**DB69_length_590_mean_cov_11.793220339**

```
AGTATTTACACCAGCCCTTGGAGATTTTATTAGAATCATGATTCACAGATGAGAAATTTTCCTCTTCTACAGGATTTAGTAACTAGAGT
 >chr7:116909867-116910175 - E=1e-164
AAGAATTACTCAGCAGACTGTCTATACAGACAAATGAATTCGAATATTTCCTTTGGCTATTAACCATTCATTGGCAAATTTCTTTTAAT

TTTTCTGAACCAACGGAATGAGCAAGTCAAGATGATTCCCCTGATCTATATATTGTAGACAGATAATTAAACAGTTTCTGAAAATTATT

ATTGTTCTGTCATACAGATATGGGACTTCAACACAGCAGTG|TTCATCCCAGCTTCAGGGAGAGAAGCGAAGCAATGAGTTGAGAATCA
                                           >chr7:116963449-116963731 - E=5e-158
TCTCTGGATTCTTGTATCCCATGCATAGTAATCTCCTTATCCCCTGGCCCCCTTCCTCGTTTCCTCACATTGCACGCTCAGGGACTTGT

TTGCCAGCGGATGGCCTCGGCAATCCGGAACGCACGCTCCGAGAGCCCACGGATGCTCTTTGGCCTGGAGCTTCCCTAAAGGTTCCTGT

ATTCGCGTGTGCTCGTAACCATGCAGCGATGTTCCCCCTTCCCCGCCTCACCTCATC
```
